# Supplementary material for: Identification of photoperiod-induced specific miRNAs in the adrenal glands of Sunite sheep (Ovis aries)
Source: Front Vet Sci. 2022 Jul 22;9:888207. doi: 10.3389/fvets.2022.888207 (PMC9354845; doi:10.3389/fvets.2022.888207)
Supplement: Supplementary file 1 [file Data_Sheet_1.ZIP › Supplementary file/miRDeep2 file.zip!/miRDeep2 file/Know_Structure/SP42A2/oar-mir-369.pdf]

miRBase precursor : oar-mir-369
Total read count : 16232
oar-miR-369-5p read count : 1477
oar-miR-369-3p read count : 14753
remaining reads : 2

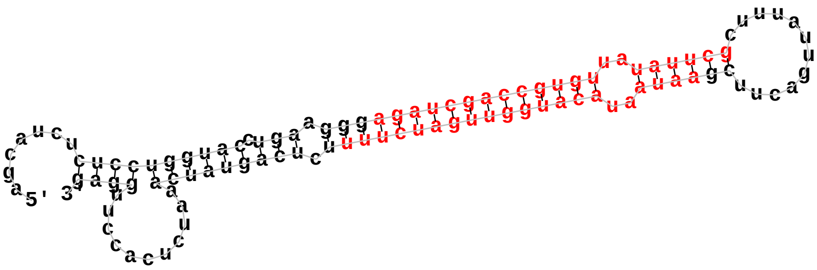

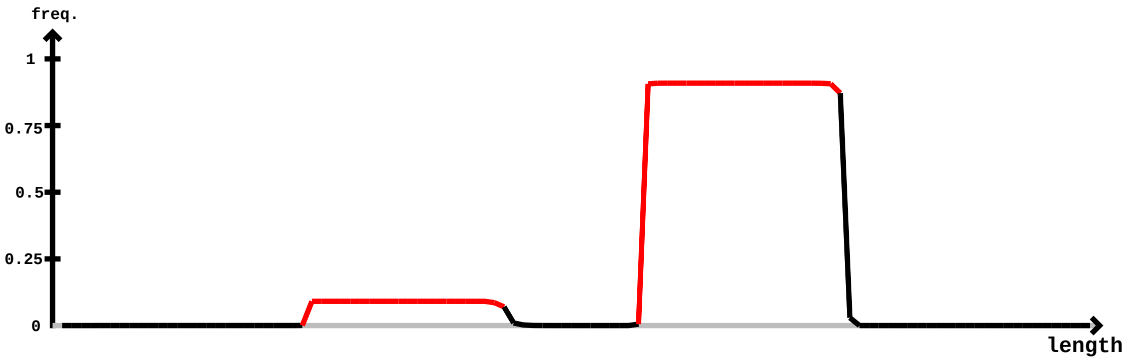


```
                                oar-miR-369-5p
                                                                oar-miR-369-3p
5'- agcaucucuccugguaccugaagggagaucgaccguguuauauucgcuuuauugacuucgaauaauacaugguugaucuuuucucaguaucaaaucucaccuuggag -3'   exp
    .......(((((((((.(((.(((((((((((((((((..((((((............))))))..))))))))))))))))).))))))))...........))))    reads   mm   sample
    ...aucucuccugguaccugaaggg..................................................................................    1       0    seq
    ......ucuccugguaccugaaggg..................................................................................    1       0    seq
    .......................gagaucgaccguguuauauucg.............................................................    4       0    seq
    .........................agaucgaccguguuaua.................................................................    1       0    seq
    .........................agaucgaccguguuauauu...............................................................    83      0    seq
    .........................agaucgaccguguuauauuc..............................................................    252     0    seq
    .........................agaucgaccguguuauauucg.............................................................    978     0    seq
    .........................agaucgaccguguuauauucgc............................................................    120     0    seq
    .........................agaucgaccguguuauauucgcu...........................................................    31      0    seq
    .........................agaucgaccguguuauauucgcuu..........................................................    5       0    seq
    .........................agaucgaccguguuauauucgcuuu.........................................................    2       0    seq
    ..........................gaucgaccguguuauauuc..............................................................    1       0    seq
    ..........................................................cgaauaauacaugguugaucuuu..........................    1       0    seq
    ...........................................................gaauaauacaugguugaucu............................    1       0    seq
    ...........................................................gaauaauacaugguugaucuu...........................    7       0    seq
    ...........................................................gaauaauacaugguugaucuuu..........................    81      0    seq
    ............................................................aauCauacaugguugau..............................    1       1    seq
    ............................................................aauaauacaugguugauc.............................    6       0    seq
    ............................................................aauaauacaugguugaucu............................    28      0    seq
    ............................................................aauaauacaugguugaucuu...........................    562     0    seq
    ............................................................aauaauaAaugguugaucuu...........................    2       1    seq
    ............................................................aauaauacaugguugaucuuu..........................    13566   0    seq
    ............................................................aauaauacaugguugaucuuuu.........................    456     0    seq
    ............................................................aauaauacaugguugaucuuuuc........................    1       0    seq
    .............................................................auaauacaugguugaucuuu..........................    21      0    seq
    .............................................................auaauacaugguugaucuuuu.........................    17      0    seq
    ..............................................................uaauacaugguugaucuu...........................    1       0    seq
    ..............................................................uaauacaugguugaucuuu..........................    2       0    seq
```
